# Response to “Letter in response to ‘Students’ age and parental level of education influence COVID-19 vaccination hesitancy’”

**DOI:** 10.1007/s00431-022-04432-9

**Published:** 2022-04-19

**Authors:** Anna Zychlinsky Scharff, Mira Paulsen, Paula Schaefer, Fatma Tanisik, Rizky Indrameikha Sugianto, Nils Stanislawski, Holger Blume, Bernhard M. W. Schmidt, Stefanie Heiden, Meike Stiesch, Anette Melk

**Affiliations:** 1 Department of Pediatric Hematology and Oncology, Hannover Medical School, Hannover, Germany; 2 Department of Pediatric Kidney, Liver, and Metabolic Diseases, Hannover Medical School, Carl-Neuberg-Str. 1, 30625 Hannover, Germany; 3 Department of Prosthetic Dentistry and Biomedical Material Research, Hannover Medical School, Hannover, Germany; 4 Institute of Microelectronic Systems, Leibniz University Hanover, Hannover, Germany; 5 Department of Nephrology and Hypertension, Hannover Medical School, Hannover, Germany; 6 Institute of Innovation Research, Technology Management & Entrepreneurship, Leibniz University Hanover, Hannover, Germany

To the Editor,

We thank the authors of the Letter to the Editor for their interest in our work and appreciate the opportunity to reply. Firstly, Sakatsume and O’Shea assert that by reporting on the percentage of students who had already received a COVID-19 vaccination, a “severe bias” may have been introduced into the study. We respectfully disagree: already vaccinated individuals were not included in the mixed model analysis examining the yes/no answer to the intention-to-vaccinate questionnaire. The figure in our report clearly delineates which study participants were vaccinated and which were not, and excludes the already-vaccinated from the analysis of vaccine hesitancy. Furthermore, we contend that failing to report these numbers, i.e., excluding vaccinated students from the study, would itself introduce bias. Therefore, we report vaccination status as well as intention-to-vaccinate to clearly denote these separate groups in our report.

Secondly, we agree that examination of students’ education levels would be a relevant and interesting approach. We examined two secondary schools with different levels of education: one was a “Gymnasium”, which is the most academically advanced among the three types of German secondary schools and primarily prepares students for university. The second school was a “Gesamtschule”, which combines the three types of German secondary schools and enables students to learn at an appropriate individual level, preparing for an academic as well as non-academic vocation depending on the individual graduation level. Because the later school included a heterogeneous population, we could not draw direct comparisons between the two schools — some students at the Gesamtschule received a very similar education to those at the Gymnasium while others received a more vocational, work-experience-oriented curriculum. Indeed, the categorization of German secondary schools by academic strength is increasingly complex and blurry as a movement away from early sorting has taken place. Regardless, we enthusiastically endorse the call by Drs. Sakatsume and O’Shea for more data on student education level and vaccine hesitancy in future studies.

Lastly, Drs. Sakatsume and O’Shea perceived discrepancies in the study population. To clarify, the longitudinal study we conducted began with one school in June 2020, and a second school was added in November 2020. In Paulsen et al. [1], we report longitudinal data from the first school, as this was the only institution for which we had already gathered a year’s worth of data. Since Paulsen et al. [1] examines changing viewpoints over time, returning to the same set of students regularly was paramount. As our paper seeks to quantify vaccine hesitancy at one specific time point, we included all the study participants at that time point — from both schools — in the current analysis. The number of students included as well as the fact that they attend two schools is reported in the “Methods”.

Vaccine hesitancy is a crucial issue in controlling future epidemics, and we thank Drs. Sakatsume and O’Shea for raising their points openly to promote the discussion of this important topic.

Sincerely,

Anna Zychlinsky Scharff, Mira Paulsen, Anette Melk.
